# Chromatin interactions reveal novel gene targets for drug repositioning in rheumatic diseases

**DOI:** 10.1136/annrheumdis-2018-214649

**Published:** 2019-05-15

**Authors:** Paul Martin, James Ding, Kate Duffus, Vasanthi Priyadarshini Gaddi, Amanda McGovern, Helen Ray-Jones, Annie Yarwood, Jane Worthington, Anne Barton, Gisela Orozco

**Affiliations:** 1 Lydia Becker Institute of Immunology and Inflammation, Faculty of Biology, Medicine and Health, University of Manchester, Manchester, UK; 2 Centre for Genetics and Genomics Versus Arthritis, Centre for Musculoskeletal Research, Manchester Academic Health Science Centre, University of Manchester, Manchester, UK; 3 Manchester University NHS Foundation Trust, Manchester Academic Health Science Centre, NIHR Manchester Biomedical Research Centre, Manchester, UK

**Keywords:** rheumatic diseases, GWAS, functional genomics, drug repositioning

## Abstract

**Objectives:**

There is a need to identify effective treatments for rheumatic diseases, and while genetic studies have been successful it is unclear which genes contribute to the disease. Using our existing Capture Hi-C data on three rheumatic diseases, we can identify potential causal genes which are targets for existing drugs and could be repositioned for use in rheumatic diseases.

**Methods:**

High confidence candidate causal genes were identified using Capture Hi-C data from B cells and T cells. These genes were used to interrogate drug target information from DrugBank to identify existing treatments, which could be repositioned to treat these diseases. The approach was refined using Ingenuity Pathway Analysis to identify enriched pathways and therefore further treatments relevant to the disease.

**Results:**

Overall, 454 high confidence genes were identified. Of these, 48 were drug targets (108 drugs) and 11 were existing therapies used in the treatment of rheumatic diseases. After pathway analysis refinement, 50 genes remained, 13 of which were drug targets (33 drugs). However considering targets across all enriched pathways, a further 367 drugs were identified for potential repositioning.

**Conclusion:**

Capture Hi-C has the potential to identify therapies which could be repositioned to treat rheumatic diseases. This was particularly successful for rheumatoid arthritis, where six effective, biologic treatments were identified. This approach may therefore yield new ways to treat patients, enhancing their quality of life and reducing the economic impact on healthcare providers. As additional cell types and other epigenomic data sets are generated, this prospect will improve further.

**Key messages**

**What is already known about this subject?**

- There is a need to identify effective treatments for rheumatic diseases.
- Selecting drug targets with genetic evidence support can double the chance of success in clinical development.
- Genetic studies, while successful, have had limited impact in identifying genes and functional mechanisms which could contribute to treatment repositioning.

**What does this study add?**

- This study provides an approach, using Capture Hi-C data, to link genetic associations to potential causal genes and assess the potential of drug repurposing in three rheumatic diseases.

**How might this impact on clinical practice or future developments?**

- This has the potential to identify genes which are functionally relevant and the target of existing therapies, which could provide new ways to treat patients, enhancing their quality of life and reducing the economic impact on healthcare providers.

## Introduction

Autoimmune rheumatic diseases such as rheumatoid arthritis (RA), juvenile idiopathic arthritis (JIA) and psoriatic arthritis (PsA) constitute a substantial socioeconomic burden estimated to cost more than €200 billion per year in Europe.^1^ While a number of therapies are used to treat symptoms, around half of all patients fail to respond well, and currently there is no cure, in part due to the lack of understanding of the biology underlying the disease. Furthermore, biologics, the most effective drugs to treat these rheumatic diseases, are expensive and constitute a major burden for healthcare systems. Indeed, the greatest overall cost of medicines in 2015/2016 in the UK was for adalimumab (£416.6 million), one of the biologics used to treat RA. Although biosimilars are emerging, the need for novel, more effective targeted treatments is therefore imperative.

For the past three decades, genetics and genomics have been incorporated into pipelines for drug discovery with the rationale that understanding the genes that cause disease may lead to a shift from alleviating symptoms to modifying the underlying mechanisms of disease.^2^ Indeed, in a review of AstraZeneca’s small-molecule drug projects from 2005 to 2010, it was found that 73% of projects with some genetic linkage of the target to the disease were active or successful in phase II compared with 43% of projects without such data.^3^ In addition, an extensive study has shown that selecting a drug target with direct genetic evidence supporting its role can double the chance of a drug’s success in clinical development.^4^

In this regard, well-powered genome-wide association studies (GWAS) have successfully identified hundreds of single nucleotide polymorphisms (SNPs) that predispose to rheumatic diseases.^5^ Some of these findings have sparked the successful repositioning of drugs, for example the association of genes in the interleukin (IL)-23 pathway; biologic drugs targeting components of this pathway are now used routinely for psoriasis and PsA and have been shown to be effective in the treatment of ankylosing spondylitis and inflammatory bowel disease.^6–8^ It has been proposed that the annual sales of these medications alone are likely to be greater than the total amount spent on GWAS in the past decade.^9^ GWAS discoveries have also highlighted novel therapeutic targets, and several programmes are currently under way to develop drugs based on this evidence, for example protein arginine deiminase inhibitors in RA.^10 11^

Despite these successful examples, the use of GWAS findings in drug discovery programmes has been quite limited. This is due to the fact that, although GWAS have identified numerous genetic variants that predispose to disease, around 90% lie outside traditional protein coding regions of the genome, often at considerable genomic distances from annotated genes,^12 13^ and therefore their potential role in pathological mechanisms is not obvious.^14 15^

Recently, functional genomics studies including chromosome conformation capture-based methods^16^ have provided evidence that complex diseases might result from a dysregulated interplay between enhancers containing disease-associated SNPs and their target genes.^17–21^ For example, in previous studies, we used Capture Hi-C (CHi-C) to characterise the chromatin interactions between all the regions of the genome associated with RA, JIA and PsA and their potential targets,^20^ and then showed that an autoimmunity variant in the 6q23 chromosomal region regulated the closest gene, *TNFAIP3*, and *IL20RA* and *IFNGR1*. Interestingly, *IL20RA* is the target of an existing drug for RA, and therefore this shows how functional evaluation of disease risk loci can help translate GWAS findings into biologically meaningful mechanisms of disease and can validate therapeutic targets or suggest new ones.^22^

In this study, we aimed to systematically mine these existing chromatin interaction data^20^ and integrate publicly available gene expression and epigenetics data to link GWAS variants to their potential target genes through physical contact. This has the potential to produce a more accurate disease gene list than simply annotating disease variants with genes in the traditional way, and therefore could identify potentially causal genes that are targets for existing drugs, which could be repositioned for use in RA, JIA and PsA.

## Methods

### Capture Hi-C

CHi-C data were produced as part of a previous larger study targeting regions associated with four autoimmune diseases (RA, JIA, PsA and type 1 diabetes)^20^ and analysed using Capture Hi-C Analysis of Genomic Organisation (CHICAGO) (online supplementary methods).^23^

10.1136/annrheumdis-2018-214649.supp1 Supplementary data

### Evaluation of reported GWAS genes

Reported GWAS hits are typically labelled according to the nearest genes; these designations were taken from the parent publications^24–27^ used for the original CHi-C study and compared with the CHi-C genes showing interactions with the SNPs linkage disequilibrium (LD) block.

### CHi-C filtering

**Figure 1:**
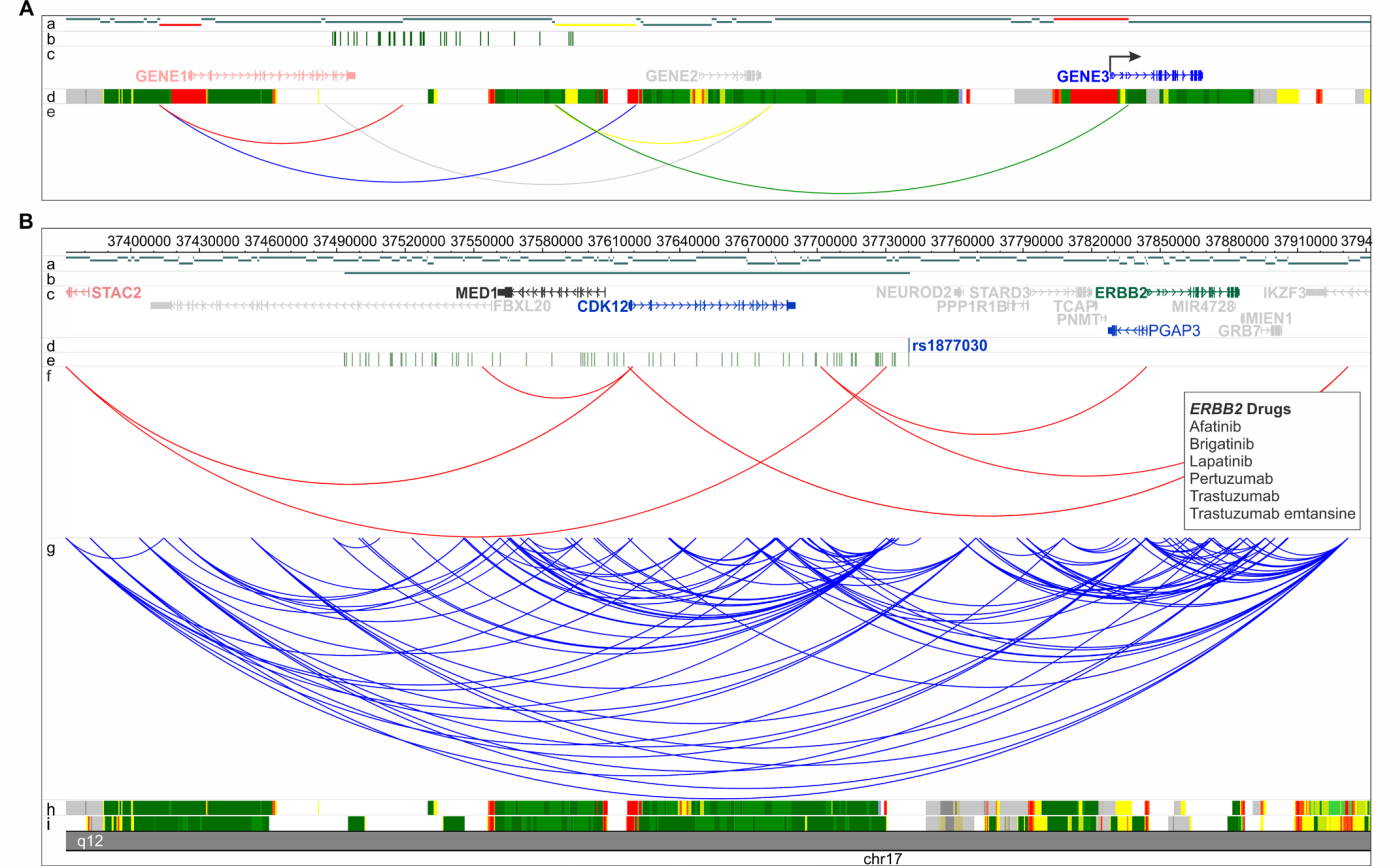
(A) CHi-C filtering strategy schematic. Tracks are labelled a–e: (a) HindIII restriction digest fragments. Promoter fragments are shown in red and enhancer fragments are shown in yellow. (b) SNPs in LD with index SNP. (c) Genes in the region. Genes showing no evidence of interacting or show no promoter and enhancer activity are shown in grey; CHi-C-filtered genes not expressed are shown in red; CHi-C-filtered genes which show evidence of promoter and enhancer activity and are expressed (arrow) are shown in blue. (d) Example 15-state ChromHMM states. (e) All CHi-C interactions within the region. Only interactions which are between promoter and enhancer fragments and are expressed (green) are used for further analysis. Interactions showing no promoter or enhancer states (grey), only promoter (red), only enhancer (yellow), or involving genes which are not expressed (blue) are removed. Therefore, using this filtering strategy, only GENE3 would be retained for further analysis. (B) Example of CHi-C region showing RA locus rs1877030. For this locus CHi-C did not identify the GWAS-reported gene, MED1, as a potential candidate, but did identify three other genes (CDK12, ERBB2 and Pgap3), one of which is a known drug target (ERBB2). Genomic coordinates are shown along the top of the region and tracks are labelled a–i: (a) HindII restriction digest fragments. (b) rs1877030 linkage disequilibrium region (r^2^ ≥0.8). (c) RefSeq genes from the UCSC Genome Browser, downloaded 1 January 2012. The GWAS-reported gene is shown in black; genes showing no evidence of interacting with rs1877030 are shown in grey; CHi-C-identified genes not expressed in B cells or T cells are shown in red; CHi-C-identified genes which are not drug targets are shown in blue and CHi-C-identified genes which are drug targets are shown in green. (d) rs1877030 location. (e) SNPs in LD with rs1877030 (r^2^ ≥0.8). (f) CHi-C interactions filtered to retain those between fragments showing enhancer states on one end and promoter states on the other end. (g) All CHi-C interactions from T cells and B cells (unfiltered). (h,i) 15-state ChromHMM states for B cells and T cells, respectively. Identified drug targets and drugs are shown in boxes. CHi-C, Capture Hi-C; GWAS, genome-wide association studies; LD, linkage disequilibrium; RA, rheumatoid arthritis; SNP, single nucleotide polymorphism; UCSC, University of California Santa Cruz.

ChromHMM chromatin state models for T helper naive, T helper memory and GM12878 lymphoblastoid cells from the Roadmap Epigenomics Project were used to filter CHi-C interactions between fragments showing enhancer states on one end and promoter states (transcription start site (TSS)) on the other end. Genes contained within the promoter state fragment (other end) were extracted and used for further analysis. Furthermore, gene lists were filtered to include only those genes expressed in either GM12878 or primary T cells (figure 1A and online supplementary methods).^28^

### Identification of drug targets

Gene lists identified during CHi-C filtering were compared against existing drug targets from the DrugBank V.5.0.11 database (https://www.drugbank.ca/releases/5-0-11). Current treatments were identified by the presence of the relevant disease name (eg, rheumatoid arthritis) and variations (eg, juvenile arthritis) in the ‘indication’ field.

### Refinement of drug targets using pathway analysis

Given that not all genes identified through the filtering step will be involved in the disease due to resolution limitation (~7 kb) of CHi-C, the approach was refined further. It was reasoned that genes belonging to enriched pathways, defined by Ingenuity Pathway Analysis (IPA), would be more likely to be involved in the disease and therefore more likely candidates. Enriched IPA pathways were identified using the core expression analysis method which uses Fisher’s exact test to identify enriched pathways, followed by Benjamini-Hochberg false discovery rate (FDR) controlling procedure to account for multiple testing. Additional targets on the same enriched pathway could also be identified and evaluated using this revised approach, expanding the list of possible drugs available for repositioning.

## Results

### Evaluation of reported GWAS genes

**Figure 2:**
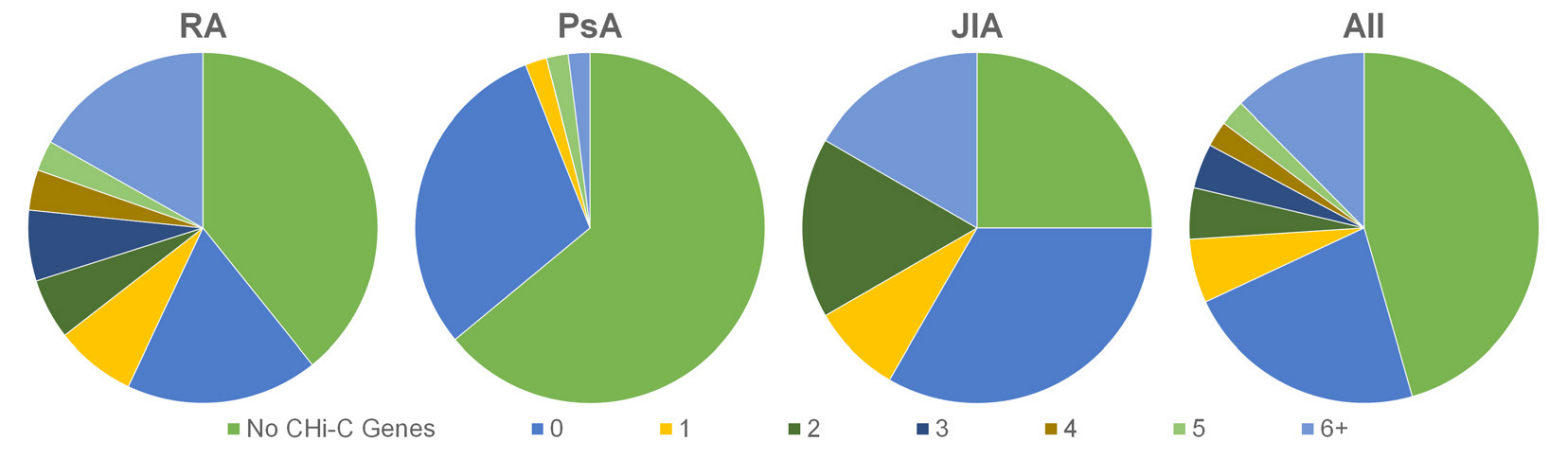
Comparison of genes identified for each SNP association loci by CHi-C and GWAS reported genes, by disease (RA, PsA, JIA) and overall (all). SNP associations where no genes were identified by CHi-C are labelled as ‘no CHi-C genes’; category 0 shows SNP associations where CHi-C identified one or more genes but where the reported gene was not among them; category 1 shows SNP associations where CHi-C only identified the reported gene and none others; and categories 2–5 and 6+ represent the number of CHi-C genes identified where the reported gene was among those identified. CHi-C, Capture Hi-C; GWAS, genome-wide association studies; JIA, juvenile idiopathic arthritis; PsA, psoriatic arthritis; RA, rheumatoid arthritis; SNP, single nucleotide polymorphism.

Our stringent filtering of the original CHi-C data identified potential causal genes for 54% of GWAS regions across all diseases, around 40% of which did not include the previously annotated causal gene (figures 1B and 2 and online supplementary table S1). Where the previously annotated gene was identified (32%), CHi-C showed evidence implicating the involvement of more than one causal gene for 44 out of 54 (81%) regions and more than five genes in 39% of cases, in addition to the previously annotated gene. This effect was largely driven by RA, which showed evidence for multiple genes at 38 out of 46 loci (83%) and more than 5 genes in 18 (39%).

10.1136/annrheumdis-2018-214649.supp2 Supplementary data

### Identification of drug targets

Overall, ChromHMM enhancer regions containing associated SNPs showed evidence of interacting with 408 genes which were expressed in either B cells or T cells, corresponding to 92 associations (54%) (table 1). Of these, 48 were existing approved drug targets, identified in DrugBank, for which 108 drugs are available (online supplementary tables S2-S4). CHi-C identified the most genes for RA (324), the most drugs (84) and 9 existing therapies used in the treatment of RA, 6 of which are effective biologic therapies (adalimumab, etanercept, rituximab, sarilumab, tocilizumab and tofacitinib). Thus 97 currently available therapies, 75 of which were for RA, not used in these diseases, were identified which could potentially be repositioned to provide effective treatment alternatives. By comparison, using the same method with RA reported genes from Okada *et al* 26 instead of CHi-C-identified genes, 24 existing drug targets were identified, corresponding to 50 drugs including 7 currently used to treat RA.

**Table 1:** Number of drug target genes identified using disease associations interacting with CHi-C genes for each disease and the corresponding number of drugs

Disease	Genes identified by CHi-C (n)	Genes which are existing drug targets (n)	Drugs identified (n)	Drugs currently used (n)	Drugs for potential repositioning (n)
RA	324	39	84	9	75
PsA	110	10	28	1	27
JIA	37	3	8	0	8
All	408	48	108	11	97

Among the drugs currently in use, six effective biologic therapies (adalimumab, etanercept and rituximab (*FCGR2A*), sarilumab and tocilizumab (*IL6R*) and tofacitinib (*TYK2*)) were identified for RA, and a relatively recent treatment, apremilast (*PDE4A*), for PsA. Drugs with potential for repositioning included alemtuzumab (*FCGR2A*), natalizumab (*ICAM1*, *FCGR2A*) and daclizumab (*IL2RA*, *FCGR2A*).

CHi-C, Capture Hi-C; JIA, juvenile idiopathic arthritis; PsA, psoriatic arthritis; RA, rheumatoid arthritis.

**Table 2:** Pathways showing significant (p≤0.05) enrichment for CHi-C genes for each disease using the Benjamini-Hochberg FDR controlling procedure

Disease	Pathway	Molecules in pathway n)	Benjamini-Hochberg p value	SNP associations (n)	Interacting genes (n)	Interacting drug targets (n)	Cell type-specific significance
RA	CD40 signalling	81	7.08×10^−4^	11	9	2	Both
	iNOS signalling	45	7.08×10^−4^	7	7	2	Both
	T helper cell differentiation	73	1.38×10^−3^	8	8	3	Both
	IL-12 signalling and production in macrophages	148	4.90×10^−3^	10	10	3	Both
	Th17 activation pathway	91	4.90×10^−3^	8	8	3	Both
	Toll-like receptor signalling	76	7.76×10^−3^	7	7	0	Both
	Dendritic cell maturation	196	8.13×10^−3^	12	11	3	Both
	April-mediated signalling	39	1.05×10^−2^	6	5	0	Both
	Molecular mechanisms of cancer	789	1.05×10^−2^	15	16	5	Both
	B cell activating factor signalling	41	1.20×10^−2^	6	5	0	Both
	IL-1 signalling	91	1.23×10^−2^	7	7	1	Both
	NF-κB signalling	187	1.23×10^−2^	11	10	2	Both
	Role of JAK family kinases in IL-6-type cytokine signalling	25	1.23×10^−2^	4	4	2	Both
	Th1 and Th2 activation pathway	187	1.23×10^−2^	9	10	6	Both
	IL-10 signalling	69	1.29×10^−2^	8	6	2	Both
	ErbB signalling	106	1.86×10^−2^	8	7	3	Both
	RANK signalling in osteoclasts	104	1.86×10^−2^	8	7	2	Both
	Th1 pathway	137	1.86×10^−2^	7	8	5	Both
	TNFR2 signalling	30	1.86×10^−2^	6	4	0	Both
	4-1BB signalling in T lymphocytes	32	2.14×10^−2^	5	4	0	Both
	IL-17A signalling in airway cells	80	2.14×10^−2^	7	6	2	Both
	IL-17A signalling in fibroblasts	35	2.82×10^−2^	5	4	0	Both
	Small cell lung cancer signalling	87	2.82×10^−2^	7	6	1	Both
	Th2 pathway	152	2.82×10^−2^	7	8	4	Both
	Production of nitric oxide and reactive oxygen species in macrophages	196	3.63×10^−2^	10	9	4	Both
	LPS-stimulated MAPK signalling	95	3.80×10^−2^	7	6	2	Both
	NF-κB activation by viruses	95	3.80×10^−2^	7	6	2	Both
	IL-8 signalling	204	4.07×10^−2^	8	9	3	Both
	Role of PKR in interferon induction and antiviral response	41	4.07×10^−2^	5	4	0	Both
	STAT3 pathway	132	4.17×10^−2^	7	7	3	Both
	mTOR signalling	208	4.37×10^−2^	8	9	4	Both
	IL-6 signalling	136	4.47×10^−2^	8	7	2	GM12878
	PI3K signalling in B lymphocytes	135	4.47×10^−2^	9	7	2	Both
PsA	Role of JAK1, JAK2 and TYK2 in interferon signalling	24	4.07×10^−2^	3	3	1	
	Epithelial adherens junction signalling	149	4.47×10^−2^	4	5	1	Both
	Interferon signalling	36	4.47×10^−2^	3	3	1	GM12878
	Regulation of actin-based motility by rho	89	4.47×10^−2^	3	4	0	Both
JIA	iNOS signalling	45	3.63×10^−3^	3	3	1	Both
	Interferon signalling	36	3.63×10^−3^	3	3	1	
	Th17 activation pathway	91	1.95×10^−2^	3	3	1	GM12878
	Role of JAK1, JAK2 and TYK2 in interferon signalling	24	2.09×10^−2^	2	2	1	Both
	Role of JAK family kinases in IL-6-type cytokine signalling	25	2.09×10^−2^	2	2	1	Both
	IL-15 production	28	2.14×10^−2^	2	2	1	Both
	PI3K/AKT signalling	129	2.14×10^−2^	2	3	1	Both
	STAT3 pathway	132	2.14×10^−2^	3	3	1	Both
	Th1 pathway	137	2.14×10^−2^	2	3	2	Both
	Oncostatin M signalling	40	2.57×10^−2^	2	2	1	Both
	Role of PKR in interferon induction and antiviral response	41	2.57×10^−2^	2	2	0	Both
	Th1 and Th2 activation pathway	374	3.89×10^−2^	2	3	2	Both
	Production of nitric oxide and reactive oxygen species in macrophages	196	4.07×10^−2^	3	3	1	Both
	IL-8 signalling	204	4.27×10^−2^	3	3	1	Both

The total number of molecules in the pathway is shown together with the number of SNP associations and the corresponding numberof genes and drug targets on the pathway. ‘Cell type specific significance’ specifies whether a pathway is significant using T cell (Jurkat) only or Bcell (GM12878) only genes (online supplementary methods). ‘Both’ states that the pathway was significant in both T cell-only genes and B cell-onlygenes

CHi-C, Capture Hi-C; FDR, false discovery rate; IL, interleukin; JIA, juvenile idiopathic arthritis; LPS, Lipopolysaccharide; MAPK, mitogen-activated protein kinase; NF-κB, nuclear factor kappa-light-chain-enhancer of activated B cells; PKR, protein kinase R; PsA, psoriatic arthritis; RA, rheumatoid arthritis; RANK, Receptor activator of nuclear factor κ B; SNP, single nucleotide polymorphism; STAT3, signal transducer and activator of transcription 3; iNOS, inducible nitric oxide synthase; mTOR, mechanistic target of rapamycin.

**Table 3:** Number of drug target genes identified for CHi-C genes enriched in significant pathways (Benjamini-Hochberg) for each disease, corresponding to the number of drugs and the potential for non-existing pathway gene targets for drug repositioning

Disease	Genes identified by CHi-C (n)	Genes which are existing drug targets (n)	Drugs identified (n)	Drugs currently used (n)	Drugs for potential repositioning (n)	Potential pathway targets (n)	Potential pathway drugs (n)
RA	50	13	38	8	30	283	398
PsA	9	2	2	0	2	47	87
JIA	10	2	4	0	4	205	325
All	59	14	39	8	31	307	412

CHi-C, Capture Hi-C; JIA, juvenile idiopathic arthritis; PsA, psoriatic arthritis; RA, rheumatoid arthritis.

Among the drugs identified by CHi-C, 23% are used in the treatment of various carcinomas, lymphomas, melanomas and leukaemia, 9% in the treatment of multiple sclerosis and psoriasis, and 7% in the treatment of hypertension across all diseases (online supplementary tables S2-S4). These include alemtuzumab, used in the treatment of chronic lymphocytic leukaemia and multiple sclerosis, and natalizumab and daclizumab, used in the treatment of multiple sclerosis. Daclizumab has been trialled for the treatment of JIA-associated uveitis and resulted in five out of six participants showing a two-step reduction in inflammation (NCT00130637; https://clinicaltrials.gov/ct2/show/NCT00130637).

### Refinement of drug targets using pathway analysis

IPA of the CHi-C-identified genes resulted in 139 enriched pathways across the three diseases (online supplementary table S5). However after controlling for FDR (p≤0.05), 41 pathways remained significant (table 2 and online supplementary table S6); these included CD40 signalling, T helper cell differentiation, and the JAK1, JAK2 and TYK2 in interferon signalling pathways.

Considering only those drug targets identified through CHi-C that form part of an enriched pathway reduced the total number of genes from 408 to 59 and the number of potential drugs for repositioning to 31 (table 3 and online supplementary table S5). Additionally eight drugs currently used to treat RA were identified: six biologic therapies (adalimumab, etanercept, rituximab, sarilumab, tocilizumab and tofacitinib), one analgesic (acetylsalicylic acid) and immune globulin human. Interestingly, only 42% of these genes represented reported GWAS genes. Expanding this gene list to include all genes involved in significant pathways resulted in 307 potential pathway target genes, corresponding to 412 drugs overall. Over 92% (283 of 307) of the potential pathway target genes and 96% of the potential pathway drugs were identified using RA GWAS associations. The approach also identified 95% (57 of 60) of existing drugs overall, including 54 of 57 drugs currently used in the treatment of RA.

**Figure 3:**
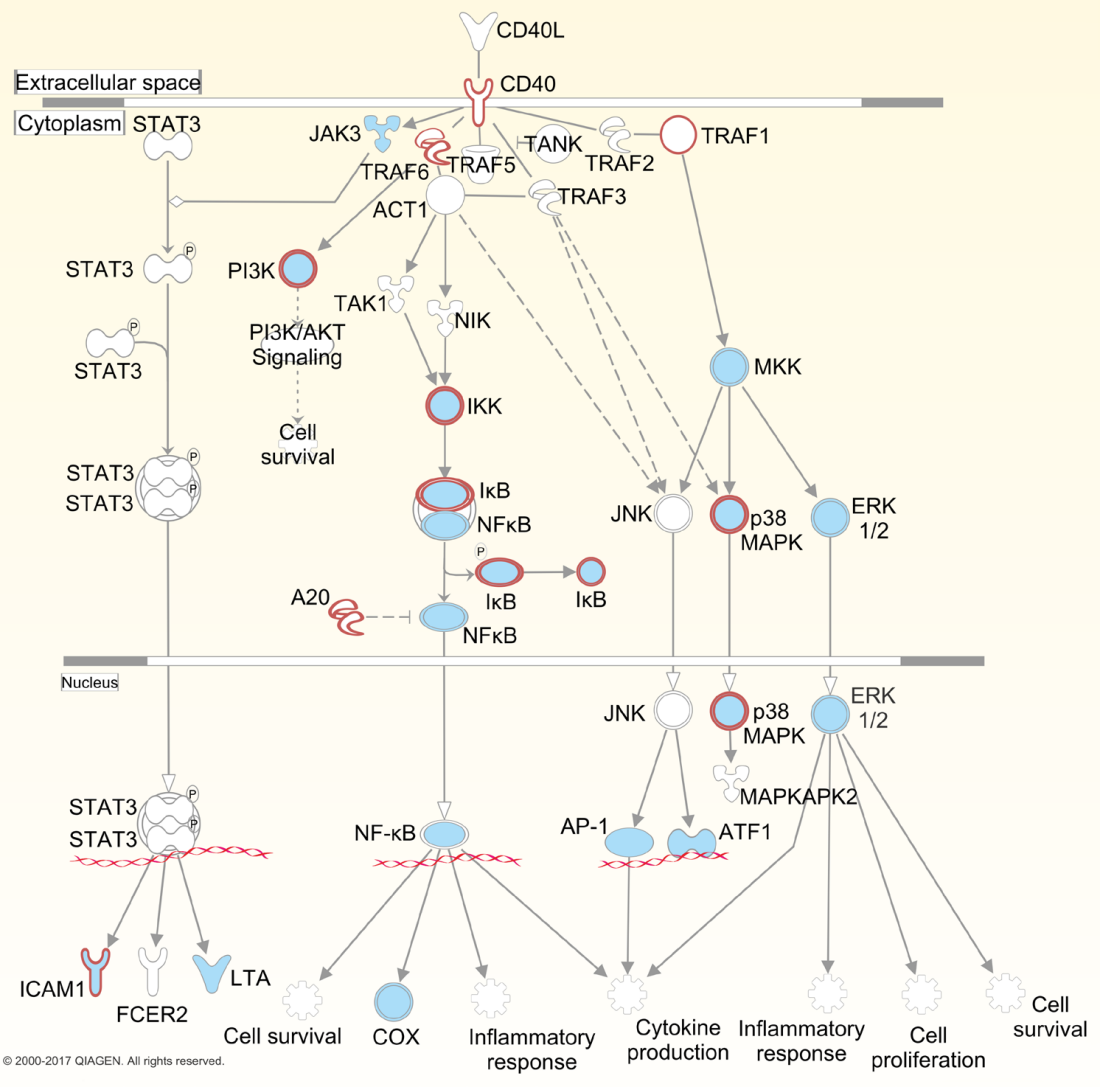
CD40 signalling pathway. Capture Hi-C-identified genes are outlined in red and existing drug targets are shaded in blue.

Figure 3 shows the CD40 signalling pathway enriched for RA CHi-C genes, identifying 9 out of 34 possible targets. Two of these, *ICAM1* and *ATM*, are existing drug targets used in the treatment of multiple sclerosis, fatigue, orthostatic hypotension and osteoarthritis (natalizumab, caffeine and hyaluronic acid, respectively) and may provide potential targets for repositioning. This pathway shows the potential for the repositioning of existing targets (shaded in blue), such as *JAK3, MKK*, and *ERK1* and *ERK2*, and the possibility of novel targets (outlined in red), such as *CD40*, *TRAF1* and *TRAF6*. Indeed, an experimental drug targeting the *CD40* receptor is currently in phase II clinical trial for the treatment of lupus nephritis, caused by systemic lupus erythematosus (NCT02770170; https://www.boehringer-ingelheim.com/press-release/phase-ii-trial-now-enrolling-patients-lupus-nephritis).

## Discussion

We had previously generated data from chromosome conformation capture experiments to improve the assignment of genes to susceptibility variants identified through GWAS based on physical interactions rather than proximity for RA, JIA and PsA. For the first time, these data have been used to identify potential novel drug targets. Linking to DrugBank has allowed the identification of a number of existing drugs that could be tested for efficacy in these conditions.

The approach of using GWAS data to identify potential drug targets for complex diseases has been explored previously. In 2014, Okada *et al* assessed the potential of RA genetics in drug discovery based on the findings of a large transethic GWAS meta-analysis.^26^ Using reported GWAS genes, or genes from a direct protein–protein interaction network, they tested 2430 genes against 871 drug target genes, defined as approved, in clinical trials or experimental, and found a significant 3.7-fold enrichment for approved RA drug targets. This corresponded to 67% of the approved RA target genes included in the analysis. However, the study was limited by their initial gene list of reported GWAS genes based purely on linear proximity on the chromosome of disease SNPs and genes.

A major strength of this study is the use of physical interactions of chromatin to identify genes directly affected by disease susceptibility variants. The advantage and validity of this approach were demonstrated by the increased ability to identify existing therapies (95% of all existing RA therapies). While this is promising, the method was, however, unable to identify three currently used RA drugs: prednisolone, methylprednisolone and methotrexate. Both prednisolone and methylprednisolone are corticosteroids which target the glucocorticoid receptor (*NR3C1*), which is located on 5q31.3, over 10 megabases from the nearest association, while the gene target for methotrexate (*DHFR*) is located over 15 megabases from the nearest association. The action of these drugs is therefore unlikely to be due to any disease susceptibility association; however, the efficacy of the drugs may still be influenced by genetics as previously shown.^29^

While other methods exist which predict enhancer targets, such as integrated method for predicting enhancer targets (IM-PET),^30^ CHi-C has the potential to directly link disease associations to causal genes by interrogating the physical interactions between these regions. These interactions have been shown to play a role in gene regulation and can therefore link disease enhancers to target genes. Additionally, using this method, we have shown evidence that one disease association may well affect more than one gene, something which is often not considered. There are, however, limitations to this approach dependent first on the completeness of the mapping of disease variants for a particular disease and second on the choice of cell type for generation of the chromosome conformation capture data. In this study the genetic architecture was better mapped for RA than for JIA or PsA, and the choice of cell type had a stronger evidence base for RA, potentially limiting the power of the analysis for PsA and JIA.

Due to the high degree of overlapping interactions, and therefore genes, between cell types and the shared protein functions (mainly general immune signalling-related proteins), many of the IPA pathways were significantly enriched in both cell types individually and therefore cannot explicitly implicate a cell type directly. However, certain pathways exhibited a higher significance in one cell type; for example, the IL-6 signalling and role of JAK family kinases in IL-6-type cytokine signalling pathways were both more associated in B cell-only genes, validating the approach. The integration of other epigenetic resources may help to further implicate a particular cell type and inform drug choice.

Furthermore, while most CHi-C interactions are shared between cell types, the failure to identify a putative causal gene for just under half of all disease regions may be due to the cell types studied as the associated variant may exhibit its effect in different cell types such as fibroblast-like synoviocytes, the major cell type present in inflamed joints. The selection of additional cell types, for each disease, should be informed by existing data: for example, while B cells and CD4+ T cells are important cell types in PsA, the decreased CD4+:CD8+ T cell ratio, the significant association with HLA-B*27 and the enrichment of associations to CD8+ H3K4me3 peaks suggest that CD8+ T cells are a major cell type in PsA,^27 31^ and therefore the genes and/or interactions mediated by the associated variants may not fully be observed in this study. This may also explain the higher proportion of SNP associations where CHi-C was unable to identify any genes for PsA, compared with RA and JIA (figure 2). A limitation could also be the use of cell lines rather than primary cells for the generation of CHi-C data. This has implications, both in terms of the cells genetic and epigenetic profiles, which could influence the presence or strength of an interaction, confounding the results. Further experiments would therefore need to be performed in multiple, genetically diverse patient samples with integration of additional genetic and epigenetic data to control for this. Despite this, CHi-C provided evidence that almost half (49%) of all disease associations may either be incorrectly annotated or represent multiple potential gene targets in addition to the previously reported gene. CHi-C was more successful in identifying genes for RA, identifying potential target genes for over 60% of associations.

While these findings are promising and we have applied strict filtering of significant interactions, it should be noted that GWAS-based omics enrichment analyses can be biased towards false positives. However, due to the experimental aim of the initial CHi-C experiment, to target and investigate interactions between GWAS loci for the three rheumatic diseases (RA, PsA and JIA), it is not possible to fully correct for this. Further whole-genome CHi-C or validation experiments would assist in controlling for this limitation.

Additionally, while DrugBank provides the most comprehensive and accessible drug target resource, the exploration and integration of multiple drug target databases may identify additional, potentially useful drugs or help prioritise our existing drug targets. While our approach is unique in using CHi-C data to identify potential target genes, other target-searching methods, such as Open Targets Platform, exist.^32^ Despite offering complementary approaches, the integration of these resources may further refine and prioritise any potential drug target candidates.

The experimental approach could be further refined and improved as, while CHi-C provides support for an involvement of a gene in disease, it is important to consider the effect of a variant in disease: for example, whether the gene is upregulated or downregulated and what implication this has on a drug’s mode of action. It would therefore be important to incorporate gene expression data which would provide this information. Furthermore, studies using the CRISPR-Cas9 genome editing system would provide an independent validation of the associated variant effect at a pathway level and test the result of perturbations to this pathway.

Using existing data, we have evaluated the potential of CHi-C to identify new therapeutic targets or existing drugs which could be repurposed to treat rheumatic diseases. It is, however, important that any drugs identified by this or similar approaches are presented in full and evaluated extensively by biopharmaceutical industry professionals to confirm their potential for repurposing. It should also be noted that, despite this, not all drugs identified using these approaches will be successfully repurposed. For example, alemtuzumab, natalizumab and daclizumab, identified during this study, have been trialled for use in RA, but either showed little benefit (alemtuzumab and natalizumab) or did achieve Food and Drug Administration approval (daclizumab),^33^ but was withdrawn due to unacceptable side effects including liver damage, encephalitis and meningoencephalitis.^34^ This illustrates that while genetic evidence can provide support for drugs targets, trials are clearly required to confirm efficacy and safety.

Drug development is an expensive and time-consuming procedure, costing pharmaceutical companies an estimated $2.6 billion per drug and taking at least 10 years to develop. Additionally about 9 out of 10 drugs fail to make it to market either due to lack of efficacy or unacceptable adverse events.^35 36^ Using genetics to inform drug development has the potential to dramatically cut costs and improve the likelihood of success. Drug repositioning is a complementary approach that removes the time spent on drug development and safety considerations entirely, and instead allows the drug to proceed straight to a tolerance/efficacy clinical trial.

We have therefore shown that CHi-C has the potential to identify existing drug targets which could be repositioned to treat rheumatic diseases. This was particularly successful for RA, where six effective, biologic treatments were initially identified, followed by 95% of existing therapies. Furthermore, while this analysis was limited to the three diseases selected (RA, PsA and JIA), this approach could be applied to other disease-specific or whole-genome promoter CHi-C data sets to identify potential drugs for further diseases. As more is known about the genetic component of PsA and JIA, coupled with further, more relevant CHi-C and epigenomic data sets, this approach may yield new ways to treat patients with rheumatic diseases, enhancing their quality of life and reducing the economic impact on healthcare providers.
