# Supplementary material for: Chromatin interactions reveal novel gene targets for drug repositioning in rheumatic diseases
Source: Ann Rheum Dis. 2019 May 15;78(8):1127–34. doi: 10.1136/annrheumdis-2018-214649 (PMC6691931; doi:10.1136/annrheumdis-2018-214649)
Supplement: Supplementary data [file annrheumdis-2018-214649supp001.docx]

## SUPPLEMENTARY METHODS

### Capture Hi-C

Briefly, all promoters within 1Mb of associated SNPs were selected and RNA baits were designed to the ends of all fragments within 500bp of the transcription start sites. Separately, associated regions were defined by SNPs in LD (r^2^≥0.8) and all restriction fragments not selected for the promoter capture experiment were targeted. Experiments were performed using human T-cell (Jurkat) and B-cell (GM12878) lines. CHi-C libraries were sequenced using 75bp paired-end reads on an Illumina HiSeq 2500. Resulting reads were mapped to restriction fragments and filtered using the Hi-C User Pipeline (HICUP http://www.bioinformatics.babraham.ac.uk/projects/hicup). Chromatin interactions were analysed using CHiCAGO (Capture Hi-C Analysis Of Genomic Organisation, http://regulatorygenomicsgroup.org/chicago), a publicly available, open-source, bespoke statistical model for detecting significant interactions in CHi-C data at a single restriction fragment resolution.[1] CHiCAGO corrects for background signal using a two-component convolution model and implements a weighted false discovery control procedure that specifically controls for false interactions at smaller distances.

### Capture Hi-C Filtering

Expanded 18-state ChromHMM models for T helper naive (E038), T helper memory (E037) and GM12878 lymphoblastoid cells (E116) were downloaded from the Roadmap Epigenomics project website (<http://egg2.wustl.edu/roadmap/data/byFileType/chromhmmSegmentations/ChmmModels/core_K27ac/jointModel/final/>) and assigned subsets according to enhancer states (chromatin states 7-11) and TSS states (chromatin states 1-4).[2] The T helper naïve and T helper memory were merged to produce ‘T-cell’ enhancer and promoter lists. These lists were intersected with the relevant cell type specific CHi-C interactions and interactions were filtered to retain those between fragments showing enhancer states on one end and promoter states (TSS) on the other end. Genes contained within the promoter state fragment (other end) were extracted and used for further analysis. Furthermore, gene lists were filtered to include only those genes expressed with a TPM ≥ 1 in either GM12878 (https://www.encodeproject.org/files/ENCFF315WZE/) or primary T-cells (https://www.encodeproject.org/files/ENCFF158VJT/) (Figure 1a).[3] A complete list of CHi-C identified enhancers, promoters, drugs and overlapping GWAS catalogue variants[4] are shown in Table S7.

### Refinement of drug targets using pathway analysis

In addition to the combined Jurkat and GM12878 gene lists, cell-type specific gene lists were also used to identify enriched IPA pathways using the core expression analysis method to identify pathways preferentially enriched in a particular cell-type.

### Supplementary References

1 Cairns J, Freire-Pritchett P, Wingett SW, *et al.* CHiCAGO: robust detection of DNA looping interactions in Capture Hi-C data. *Genome Biol* 2016;**17**:127. doi:10.1186/s13059-016-0992-2

2 Ernst J, Kellis M. ChromHMM: automating chromatin-state discovery and characterization. *Nat Methods* 2012;**9**:215–6. doi:10.1038/nmeth.1906

3 ENCODE Project Consortium. An integrated encyclopedia of DNA elements in the human genome. *Nature* 2012;**489**:57–74. doi:10.1038/nature11247

4 Welter D, MacArthur J, Morales J, *et al.* The NHGRI GWAS Catalog, a curated resource of SNP-trait associations. *Nucleic Acids Res* 2014;**42**:D1001-6. doi:10.1093/nar/gkt1229
